# Alterations of hair cortisol and dehydroepiandrosterone in mother-infant-dyads with maternal childhood maltreatment

**DOI:** 10.1186/s12888-017-1367-2

**Published:** 2017-06-06

**Authors:** K. Schury, A. M. Koenig, D. Isele, A. L. Hulbert, S. Krause, M. Umlauft, S. Kolassa, U. Ziegenhain, A. Karabatsiakis, F. Reister, H. Guendel, J. M. Fegert, I.-T. Kolassa

**Affiliations:** 1 Clinical and Biological Psychology, Institute of Psychology and Education, Ulm University, Albert-Einstein-Allee 47, 89081 Ulm, Germany; 2 Department of Psychology, University of Konstanz and vivo international, 78457 Konstanz, Germany; 3 Department of Psychosomatic Medicine and Psychotherapy, University Hospital Ulm, 89081 Ulm, Germany; 4 Institute of Statistics, Ulm University, 89081 Ulm, Germany; 5 SAP Switzerland, 8274 Tägerwilen, Switzerland; 6 Department of Child and Adolscent Psychiatry and Psychotherapy, University Hospital Ulm, 89075 Ulm, Germany; 7 Department of Obstetrics and Gynecology, University Hospital Ulm, 89075 Ulm, Germany

**Keywords:** Cortisol, DHEA, Hair, Childhood maltreatment, Pregnancy, Transgenerational

## Abstract

**Background:**

Child maltreatment (CM) has severe effects on psychological and physical health. The hypothalamic-pituitary-adrenal (HPA) axis, the major stress system of the body, is dysregulated after CM. The analysis of cortisol and dehydroepiandrosterone (DHEA) in scalp hair presents a new and promising methodological approach to assess chronic HPA axis activity. This study investigated the effects of CM on HPA axis activity in the last trimester of pregnancy by measuring the two important signaling molecules, cortisol and DHEA in hair, shortly after parturition. In addition, we explored potential effects of maternal CM on her offspring’s endocrine milieu during pregnancy by measuring cortisol and DHEA in newborns’ hair.

**Methods:**

CM was assessed with the Childhood Trauma Questionnaire (CTQ). Cortisol and DHEA were measured in hair samples of 94 mothers and 30 newborns, collected within six days after delivery. Associations of maternal CM on her own and her newborn’s cortisol as well as DHEA concentrations in hair were analyzed with heteroscedastic regression models.

**Results:**

Higher CM was associated with significantly higher DHEA levels, but not cortisol concentrations in maternal hair. Moreover, maternal CM was positively, but only as a non-significant trend, associated with higher DHEA levels in the newborns’ hair.

**Conclusions:**

Results suggest that the steroid milieu of the mother, at least on the level of DHEA, is altered after CM, possibly leading to non-genomic transgenerational effects on the developing fetus in utero. Indeed, we observed on an explorative level first hints that the endocrine milieu for the developing child might be altered in CM mothers. These results need extension and replication in future studies. The measurement of hair steroids in mothers and their newborns is promising, but more research is needed to better understand the effects of a maternal history of CM on the developing fetus.

**Electronic supplementary material:**

The online version of this article (doi:10.1186/s12888-017-1367-2) contains supplementary material, which is available to authorized users.

## Background

Child maltreatment (CM) can have detrimental psychological and biological consequences for the affected individuals and may even affect the next generation via behavioral (e.g., parenting) but probably also via genomic and non-genomic biological pathways. An elevated risk for diverse health problems in adulthood has been described in individuals with a history of CM; for example, psychopathology, cardiovascular disease, cancer, or even premature mortality [1–3]. One possible underlying mechanism between CM and later physical health problems may be the dysregulation of the hypothalamic-pituitary-adrenal (HPA) axis, the body’s major stress system. Functional HPA axis activity is essential for the regulation of metabolic processes in order to maintain physiological homeostasis, but its functioning can be dysregulated upon chronic stress such as CM, in particular when it occurs during sensitive developmental periods.

A stress-induced activation of the HPA axis leads to the release of corticotropin-releasing hormone (CRH) from the hypothalamus, which causes the secretion of adrenocorticotropic hormone (ACTH), and in turn results in the secretion of glucocorticoids (mainly cortisol) from the adrenal cortex. Besides cortisol, the steroid hormone dehydroepiandrosterone (DHEA) and its sulfate ester DHEA-S are released upon stress. There is growing interest in the investigation of both cortisol and DHEA, as their interplay impacts many physiological systems via genomic and non-genomic mechanisms, but they seem to have opposing biological, neurological and immune-related functions (for a review see [4]). Hence, taking both hormones into account may be a more sensitive index for the regulation of HPA axis activity [4].

A stress-induced increase of *cortisol* is crucial in response to environmental stressors as it results in the provision of energy by increasing the release of glucose and inhibition of non-essential functions (e.g., reproduction, growth; [4]). However, persistently altered levels of cortisol can have health-threatening effects (for more details see [5, 6]).

*DHEA* can antagonize some of the effects of cortisol [7]. Indeed, neuroprotective, antioxidant, anti-inflammatory, and immune-modulatory effects have been described for DHEA (for details on metabolic pathways and physiological function of DHEA see e.g. [8–11]). It is related to a broad range of physiological processes, as DHEA is a precursor of many other steroid hormones (e.g., testosterone, estradiol; [8–10, 12]). Both cortisol and DHEA have been suggested as biomarkers for the regulation of HPA axis activity and related to psychiatric diseases such as depression, anxiety, PTSD, dementia, eating disorder, and externalizing problems during childhood, adolescence and even adulthood (for reviews see [4, 8]).

CM as a chronic and traumatic stressor can lead to a persistent dysregulation of the HPA axis activity, resulting in altered *cortisol* secretion in affected individuals [13] and their children [14]. However, findings on long-term effects of CM on cortisol measured in blood, urine and saliva are mixed (e.g., [15–18]). By measuring cortisol in *hair* as a reliable measure of chronic HPA axis activity [19–21], several studies found an association of CM with lower levels of cortisol: an association of CM and decreased hair cortisol levels, independent of current major depression diagnosis was reported in a study with depressed patients (27 women and 16 men; mean age 41.7) and healthy age- and sex-matched controls [22]. Another study with 55 healthy college students (18 to 24 years of age) also reported a negative association of adverse childhood experiences and hair cortisol [23].

In contrast to cortisol, only few studies considered the role of *DHEA* in the aftermath of CM so far. These studies investigated CM in PTSD patients with mixed results: whereas one study observed an association of CM with elevated concentrations of DHEA in blood plasma of PTSD patients [24], another study investigating a sample of adult smokers with and without PTSD did not find a significant effect of CM on DHEA concentrations in blood serum [18]. In the aftermath of sexual trauma, PTSD patients showed significantly reduced DHEA levels compared to age-matched controls in two studies measuring steroids in saliva [25] and blood [26]. As all reported studies investigated DHEA in blood, the assessment of DHEA in hair as a cumulative measure over time might clarify the existing inconsistent findings.

In addition, CM may also impact the affected individual’s *offspring*: children of mothers with a history of CM showed higher salivary cortisol concentrations compared to children of control mothers [14]. An effect of maternal traumatic experiences in adulthood on the offspring’s HPA axis regulation was confirmed in infants of mothers living with PTSD, with lower cortisol concentrations in saliva [27] and blood serum [28] of the offspring.

Parental CM may affect the offspring’s health and development by the dynamic interplay between environmental (e.g. parenting behavior, health behavior like smoking), and biological mechanisms (including genetic, epigenetic and non-genomic imprinting via the endocrine milieu during pregnancy). The offspring’s biological constitution may be influenced during the prenatal period already, which is one of the most sensitive periods for development [29]. On the endocrine level, a dysfunctional activity of the maternal HPA axis (e.g., due to a history of CM) may influence the developing HPA axis and endocrine milieu of the fetus during gestation with persisting effects on the offspring’s HPA axis regulation and health. Indeed, during pregnancy maternal, fetal, and placental endocrine systems strongly interact in maintaining intrauterine homeostasis, ensuring maturation of vital organs in the developing fetus and the timing of parturition [30]. Whereas cortisol is essential for fetal development, as it stimulates the differentiation and maturation of vital organ systems (in particular the fetal lungs) before birth (for reviews see [30–33]), DHEA (and its sulfated form DHEAS) is critical for the physical and neural development, as it is the main source for estrogen synthesis in the placenta, which plays a pivotal role in the timing of parturition and thus for the health and survival of the newborn [30, 32]. Furthermore, DHEA is essential for brain development and is involved in neurite growth, neurogenesis, neuronal survival, and apoptosis (for reviews see [8, 9]).

Although steroidogenesis in maternal, placental and fetal compartments is interdependent (for a review see [34]), the fetal HPA axis represents a separate biological system that is worth studying, as cortisol and DHEA, whether of fetal or maternal origin, are likely to influence development, and potentially have long-term effects on HPA function.

The analysis of cortisol and DHEA in scalp hair presents a new and promising methodological approach to measure these endocrine steroids in a non-invasive manner. Contrary to measuring acute circulating levels of cortisol and DHEA in body fluids such as blood, urine, or saliva, the assessment of cortisol and DHEA concentrations in hair allows for the retrospective analysis of the total exposure to these steroids over time [19–21]. Furthermore, the analysis of steroids in hair overcomes some of the methodological limitations when using body fluids [21] such as circadian fluctuations throughout the day.

The aim of this study was to investigate the influence of CM on the regulation of the HPA axis during pregnancy by measuring cortisol and DHEA in hair samples of postpartum women shortly after parturition. The analyzed 3 cm hair segment of these mothers was assumed to reflect the last trimester of pregnancy. Following previous findings on CM-related reductions in hair cortisol levels [22, 23], we expected a negative association of the severity of maternal CM with *cortisol* concentrations in the last trimester of pregnancy measured in hair of postpartum mothers. Furthermore, we also explored an association of CM and *DHEA* levels in the last trimester of pregnancy measured in the hair of postpartum women. In addition, we tested a potential association of the mothers’ CM experiences and her offspring’s HPA axis in an explorative analysis. Cortisol and DHEA were measured in the newborn’s hair, which were assumed to reflect fetal HPA axis activity before parturition.

## Methods

### Study design and population

Mothers and their newborns were recruited in the maternity ward of the University Hospital Ulm, Germany. Exclusion criteria were maternal age under 18 years of age, insufficient knowledge of the German language, severe complications during parturition, severe health problems of mother or child, as well as maternal drug consumption or psychotic disorders. Of 1460 mothers that were invited to participate, a total of 240 gave written informed consent within six days after parturition (*median* = 2 days; range = [0; 6] days); basic socio-demographic information was assessed and mothers were screened for CM experiences using the German version of the Childhood Trauma Questionnaire (CTQ; [35]).

To ensure sufficient statistical power to detect long-term effects of maltreatment experiences, we oversampled for higher CTQ sum scores: Based on the distribution of CTQ sum scores obtained in a pilot study with *N* = 185 postpartum women (unpublished data), the CTQ scale was divided in 25% quantiles, resulting in the following value ranges: 25–28 (25%), 29–32 (50%), 33–39 (75%) and 40–125 (100% quantile). We aimed at the same number of women in each value range at a three-month follow-up (cf. below and Additional files 1, 2 and 3). As expected, the distribution of CTQ sum scores was skewed to the right. Thus, we recruited all women with CTQ sum scores ≥ 40. For every woman with a sum score ≥ 40 who participated at the three-month follow-up, we consecutively included one women in the other value ranges. To compensate for drop-outs between *t*_*0*_ and follow-up, value ranges were filled up each time when a woman dropped out at follow-up, so that slightly more women were recruited at *t*_*0*_ than in an ideal situation without drop-outs. This led to the inclusion of 112 mothers at *t*_*0*_ (28 with CTQ sum score ≥ 40; 26 in the range 33–39, 21 with scores between 29 and 32 range and 37 with scores ≤ 29), of which hair samples could be obtained from 106 mothers and 49 newborns. Samples of one mother and 19 infants had to be excluded as the hair sample weight was below the minimum of 5 mg. Mothers with any intake of medication during pregnancy with known effects on HPA axis activity (e.g. glucocorticoids) were excluded. This led to the additional exclusion of another eleven mothers (no further hair samples of newborns had to be excluded). The final sample consisted of *N* = 94 mothers and *N* = 30 newborns. Study procedures and drop-outs are described in Additional file 1. Participants received 10€ for study participation at *t*_*0*_. All procedures were approved by the ethics committee of Ulm University.

At the time of recruitment, mean age of the 94 women was 32.45 years (range = [21; 44], *SD* = 5.44 years). Gestation lasted between 37 and 42 weeks (*mean* = 39.70, *SD* = 1.37). Further sociodemographic and medical data is reported in Table 1.

**Table 1:** Descriptive data for the 94 mothers and 30 newborns

Variable	*M ± SD* *N* (%)
Maternal data (*N* = 94)	
Hair steroids	
Cortisol in pg/mg	18.89 ± 74.51
DHEA in pg/mg	4.33 ± 11.00
Medical and demographic data**:**	
Age in years	32.45 ± 5.44
Married	72 (76.60%)
German origin	83 (88.3%)
Higher school education (> 11 years of school)	55 (58.51%)
Hair treatment	34 (36.17%)
- Colored hair	18 (19.15%)
- Permanent wave	3 (3.19%)
- Dyeing	13 (13.83%)
BMI^a^	24.54 ± 4.79
Currently smoking	9 (9.57%)
Time between parturition and hair collection (days)	2.20 ± 1.16
Psychological data	
Child maltreatment (CTQ sum score)	36.29 ± 12.96
Perceived Stress (PSS4)	4.59 ± 3.05
Lifetime psychopathology	27 (28.72%)
- Major Depression	17
- Anxiety	7
- Other	12
Newborn data (*N* = 30)	
Hair steroids	
Cortisol in pg/mg	208.00 ± 122.02
DHEA in pg/mg	3.31 ± 1.70
Medical data	
Gestational age at birth^b^	39.77 ± 1.51
Birth weight in gram^b^	3328 ± 482.35

*M* = mean; *SD* = standard deviation; *N%* = relative frequency in valid percent; *BMI* = body mass index; *CTQ* = Childhood Trauma Questionnaire; *PSS4* = Perceived Stress Scale, four-item version

^a^Missing data for BMI in 35 cases

^b^Missing data in 8 newborns

Mothers and newborns who were excluded due to low weight of hair sample or medication during pregnancy did not differ from mothers and newborns included with respect to CM experiences, maternal age, weeks of gestation, infants’ birth weight, marital status, education level, or maternal perceived stress in the last month of pregnancy (all *p* > .05).

In a subsample of *N* = 42 mothers, more detailed information on adverse childhood experiences was assessed with the German version of the Maltreatment and Abuse Chronology of Exposure (MACE; [36, 37]) three months after parturition. For the interested reader, we report recruitment and data assessment, study procedure and results regarding the association of MACE scores and HPA measurements in hair of these *N* = 42 mothers and their *N* = 15 newborns in Additional files 1, 2, and 3 respectively.

### Psychological measures

#### Maltreatment load

CM was measured with the German version of the short form of the Childhood Trauma Questionnaire (CTQ; [35]). Experiences of emotional abuse, physical abuse, sexual abuse, emotional neglect, and physical neglect are assessed with five items each and rated on a five-point Likert scale. Ratings of the 25 items were summed up, with higher values indicating higher maltreatment load.

#### Covariates

The following demographic, medical and psychological data were assessed as potential confounding variables on the effect of childhood maltreatment on hair steroids: maternal age, body mass index (BMI), smoking status, hair treatment (i.e., coloring, permanent wave or dyeing) in the past three months, perceived stress during the last month (PSS4; [38]), as well as gestational age and birth weight. The prevalence of maternal lifetime psychiatric diagnoses was assessed via self-report with the question whether one had ever been diagnosed with a psychiatric disorder during one’s lifetime.

### Collection of hair and analysis of cortisol and DHEA using HPLC-coupled mass spectrometry

Hair strands were cut from the posterior vertex region of the mothers within six days after parturition as close to the scalp as possible. In order to gain enough material for analyses (> 5 mg), two to three hair strands (~ 3 mm diameter) were collected following recommendations provided by the Society of Hair Testing [39]. In the infants, two to three hair strands were obtained following the same protocol. However, as hair in newborns is sparse, hair strands were cut at locations with most hair, most often at the hairline beneath the ear. Hair samples were then wrapped in aluminum foil and stored at −20 C° until shipping to the laboratory of Prof. Dr. Kirschbaum, Dresden, Germany, for analysis. Maternal hair strands of 3 cm adjacent to the scalp were analyzed, reflecting the last trimester of pregnancy, given an average hair growth of 1 cm per month [40]. Since studies on neonatal hair growth suggested that the metabolic activity of prenatal development in the third trimester of pregnancy is reflected in neonatal hair fibers present at birth [41], the whole hair strand was analyzed in newborns. Maternal hair samples were analyzed in different batches, newborns' hair in one batch.

Washing and extraction of cortisol and DHEA were performed according to the protocol of Gao and colleagues [20]. In short, every hair segment was washed twice in isopropanol and then dried for at least 12 h. Using the same hair segment, cortisol and DHEA were extracted from non-pulverized hair in methanol at room temperature overnight. After centrifugation at 10,000 rpm for two minutes, the supernatant was used and the methanol was evaporated at 65 °C under a constant nitrogen stream. Afterwards, the dried samples were resuspended in 150 μl double-distilled water. A Shimadzu HPLC-tandem mass spectrometer (Shimadzu, Canby, OR), which was coupled to an ABSciex API 5000 Turbo ion-spray triple quadruple tandem mass spectrometer (AB Sciex, Foster City, CA) was used for the analysis.

### Statistical analyses

All statistical analyses were conducted with R 3.2.5 [42] using a 5% level of significance. For descriptive purposes, information on mean values and standard deviations (*SD*), as well as data shown in graphs are presented in original units (pg hormone/mg hair). For correlation analyses, the non-parametric Kendall’s Tau is reported as distribution of hair data is skewed.

Typical restrictions of standard linear models are normally distributed error terms and equal variances across groups. Since these assumptions were violated, standard methods would have been not valid. If the assumption of homogeneity of variance is violated, heteroscedastic regression analysis are known to stabilize the variance of regression estimates [43] and thus, were used to draw meaningful conclusions from the present data. In short, Ordinary Least Squares (OLS) models were estimated on 10^4^ bootstrap samples and the means of the generated *b’* coefficients and standard errors, as well as *t*-statistic and *F*-statistic respectively, and *p* values are reported. For bootstrapping the residuals we used the wild bootstrap technique from the fANCOVA package [44]. For more details see Liu [45] and Rana et al. [43]. Covariates (see above) for the regression models were chosen based on Akaike’s Information Criterion (AIC; [46]). No significant differences between the batches were observed in maternal cortisol data (*W* = 895.5, *p* = .22). However, maternal DHEA concentrations significantly differed between the batches (Wilcoxon rank sum tests: *W* = 668.5, *p* = .003). Thus, a dummy variable coding for the batch effect was included as a covariate in regression analyses with maternal DHEA data.

A simple plot identified one particularly high cortisol score of 706.76 pg/mg as well as one particularly high DHEA score of 90.38 pg/mg in maternal hair data. In a chi-squared test for outliers ([47]; using R Package ‘outliers’ by Komsta; [48]) these scores significantly diverged from the distribution of all other cortisol or DHEA data (cortisol: χ^2^ = 85.24, *p* < .001; DHEA: χ^2^ = 61.14, *p* < .001) and were thus excluded in correlation and regression analyses with maternal hair steroids were to prevent these scores from hampering the statistical analyses.

## Results

A total of 21 (22.34%) mothers reported at least low to moderate levels of emotional abuse, 11 (11.70%) physical abuse, 15 (15.96%) sexual abuse, 44 (46.81%) emotional neglect, and 11 (11.70%) physical neglect in childhood according to cut-off scores described for the CTQ by Bernstein et al. [49]. CTQ sum scores ranged from 25 to 103 with a mean of 36.29 (*SD =* 12.96). Descriptive data on cortisol and DHEA concentrations in hair of women during pregnancy and their newborns, maternal exposure to childhood maltreatment, as well as medical and psychological data are reported in Table 1.

Cortisol and DHEA levels were not significantly correlated with each other in women (Kendall’s *τ* = 0.04, *p* = .53) or newborns (Kendall’s *τ* = 0.13, *p* = .33). Hair DHEA concentrations in hair of mothers were significantly correlated with DHEA levels in newborn’s hair (Kendall’s *τ* = 0.30, *p* = .003). Maternal and newborns' hair cortisol concentrations were not significantly correlated (Kendall’s *τ* = 0.12, *p* = .25).

### Association of CM with cortisol and DHEA levels in the last trimester of pregnancy measured in maternal hair

A regression model with CM as an independent variable and *hair cortisol* as a dependent variable without further covariates minimized the AIC. No significant association of CM on hair cortisol was obtained (see Table 2).

**Table 2:** Summary of heteroscedastic regression analyses for variables predicting cortisol and DHEA in hair samples of mothers and their newborns

Maternal hair data				
Cortisol concentrations (*N* = 93^a^)	*b*	*SE*	*t* (91)	*p*
Childhood maltreatment (CTQ sum score)	0.12	0.15	0.72	.471
*R* ^*2*^ = .02, *F*(1, 91) = 1.08, *p* = .302				
DHEA concentrations (*N* = 93^†^)	*b*	*SE*	*t* (91)	*p*
Childhood maltreatment (CTQ sum score)	0.28	0.08	3.25	.002**
Age	0.51	0.19	2.56	.012*
Perceived stress (PSS4)	-0.58	0.37	-1.41	.16
Hair batch	2.48	2.10	1.29	.20
*R* ^*2*^ = .25, *F*(4, 89) = 6.91, *p* < .001**				
Newborns' hair data				
Cortisol concentrations (*N* = 30)	*b*	*SE*	*t* (28)	*p*
Childhood maltreatment (CTQ sum score)	2.23	1.69	1.34	.186
Maternal age	−5.12	3.88	−1.33	.190
*R* ^*2*^ = .22, *F*(2, 27) = 3.44, *p* = .047				
DHEA concentrations (*N* = 30)	*b*	*SE*	*t* (28)	*p*
Childhood maltreatment (CTQ sum score)	0.04	0.02	1.89	.070^#^
*R* ^*2*^ = .17, *F*(1, 28) = 4.79, *p* = .037*				

^#^< .10, * < .05, ** < .01, *** <. 001

^a^One outlier in DHEA, and cortisol respectively, was excluded from regression analysis

*CTQ* = Childhood Trauma Questionnaire; *PSS4* = four-item version of the Perceived Stress Scale

**Fig. 1:**
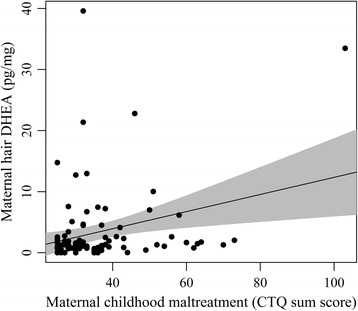
Regression line of maternal childhood maltreatment and DHEA concentrations in maternal hair, reflecting the third trimester of pregnancy. The grey area depicts the pointwise 95% confidence area

We observed a positive association of CM (CTQ sum score) and hair DHEA concentrations (*b* = 0.12; *p* = .01) controlling for batch effects in which the hair samples were analyzed (*b* = 3.49; *p* = .01). All variables explained 20% of the variance in DHEA (*F*(2, 90) = 11.66, *p* < .001). This association remained significant after controlling for maternal age and perceived stress as covariates (chosen by the AIC scores), as well as the hair batch in heteroscedastic regression (see Table 2). The full model explained 25% of the variation in hair DHEA (*F*(4, 89) = 6.91, *p* < .001). Results are illustrated in Fig. 1.

### Association of maternal CM on cortisol and DHEA levels measured in the newborn’s hair

Maternal CM was not significantly associated with prenatal cortisol levels in newborns’ hair (*b* = 2.42, *p* = .18). Including maternal age as covariate minimized the AIC. Similarly, no significant effect of the CTQ sum score on prenatal cortisol in hair of newborns was found when controlling for maternal age (see Table 2).

A main effect model of maternal CM (CTQ sum score) as an independent variable for prenatal DHEA levels measured in hair of the newborns as a dependent variable without further covariates was chosen based on the AIC for the analyses. Maternal CM was positively associated with prenatal DHEA levels in newborns, but as a non-significant trend (*b* = .04; *p* = .07). 17% of the variance in DHEA concentrations was explained by maternal CM (*F*(1, 28) = 4.79; *p* = .04). Results are shown in Table 2 and illustrated in Additional file 4.

## Discussion

In contrast to previous findings, hair cortisol levels were not associated with the amount of CM experienced in our sample of postpartum women. Prior studies reported a negative association between CM and hair cortisol [22, 23]. However, our results are in line with another study reporting no such association [50]. However, none of these studies investigated steroids during pregnancy. Differing results may be explained by the physiological and endocrine alterations in the last trimester of pregnancy, which might mask the replicated finding of lower cortisol in the aftermath of CM. In short, CRH is now also secreted by the fetal adrenal gland, the placenta and decidua, and consequently contributes to an exponential increase of maternal plasma CRH. Due to elevated CRH as well as a longer half-life of cortisol in plasma (as a result of estrogen-induced reduction of cortisol catabolism in the liver) cortisol levels increase with a peak during the third trimester of pregnancy (for more details see [33]). The one study investigating hair cortisol in relation to CM in pregnant women confirmed our results of no association in Caucasian pregnant women with relatively low levels of CM [51].

Our finding of elevated *DHEA* levels in hair is in accordance to some studies measuring DHEA in *body fluids* of PTSD patients [24, 52, 53]: Significant amounts of the variation of elevated DHEA were explained by childhood trauma history in women with PTSD [24]. However, other studies did not find an association of CM with DHEA, but with cortisol in blood plasma and serum of PTSD patients [18, 54]. Studies investigating DHEA in hair are lacking, thus further studies are needed to clarify effects of CM on chronic DHEA levels.

Furthermore, this study explored the influence of maternal CM on the next generation’s HPA axis regulation via non-genomic effects by influencing the endocrine milieu in utero of the developing fetus. In contrast to previous findings, we did not observe an association of maternal CM and cortisol levels in the newborn’s hair. However, in previous studies that related maternal CM [14] or PTSD [27, 55] with lower cortisol concentrations, cortisol was measured in saliva of the offspring at the age of six months or even adulthood. Thus, results are not comparable to our findings regarding prenatal steroid concentrations measured in newborns' hair, as prenatal HPA axis activity differs significantly from HPA axis functioning during childhood or adulthood and does not allow retrospective conclusions about the in-utero environment of the developing fetus.

Regarding *DHEA*, higher maternal CM was positively associated with elevated DHEA concentrations in the hair of our newborn sample but only as a non-significant trend (*p* = .07), explaining 17% of the variation in hair DHEA. This relationship was significant in a subsample of newborns (*N* = 15) whose mothers were interviewed in detail about a broader range of adverse childhood experiences including also witnessing physical violence towards parents or siblings, peer emotional violence, and peer physical violence (see Additional file 2). However, as the sample size of this subsample was small, this finding should be seen as hypothesis-generating for future studies, and thus no final conclusions should be drawn at this stage (see Additional files 3 and 4). The only other study investigating cumulative prenatal DHEA concentrations measured DHEA in the fingernails of newborns whose mothers had experienced stress during pregnancy and report a positive association [56]. However, they did not examine maternal lifetime stress such as adverse childhood experiences.

This results hint towards a potential effect of maternal childhood experiences on the intrauterine endocrine environment of the developing offspring in late gestation, but presumably also during the whole gestational process. Indeed, CM has been associated with altered HPA axis activity during pregnancy of affected mothers [51]; and a dysfunctional activity of the maternal HPA axis during gestation has been associated with alterations in the offspring’s HPA axis [57, 58]. Altered levels of steroids in the mother during pregnancy might influence the fetal HPA axis directly by crossing the placental barrier, and indirectly by stimulating placental CRH production and reducing uteroplacental blood flow [59]. Thus, the developing fetus might achieve and incorporate information about its later environment via intrauterine maternal-placental-fetal communication – a phenomenon referred to as “fetal programming” [60, 61]. By programming of the fetal HPA axis, the offspring may adapt to the environment in which they will be born in anticipation of an exposure to similar environmental conditions in postnatal life [57, 62]. However, results in this study regarding a potential transgenerational effect of CM were only significant on a trend level or were only present in a subsample for which also information on witnessing physical violence towards parents or siblings, peer emotional violence, and peer physical violence was available. Thus, our findings need replication in larger samples but can be used for the formulation of hypotheses and calculation of effect sizes for future studies.

### Strengths and limitations

One major limitation of this study is the relatively small sample of newborns with sufficient amount of hair for analysis due to the limited availability of sufficient hair for analysis. Studies on neonatal hair growth suggested that neonatal hair fibers at birth reflect the metabolic activity of prenatal development in the third trimester of pregnancy [41]. Nevertheless, length of maternal and newborn hair samples differed (3 cm in mothers vs. approximately 1 cm in newborns) and might not represent the exact same time period. In addition, cortisol and DHEA concentrations measured in the newborns’ hair may be of fetal and, partly, of maternal origin: Even though fetal exposure to maternal steroids is limited due to the actions of the placental 11β-HSD2 enzyme, which transforms cortisol into its inactive form cortisone and thus provides a partial barrier [59, 63, 64], a small percentage of maternal glucocorticoids still passes the placenta into the fetal system (for reviews see e.g. [65]). Furthermore, methodological issues (variability associated with growth rate of hair and inconsistencies in collection of hair samples [66, 67]) as well as incorporation of steroids in hair [68–73] are still discussed. Besides incorporation via blood during formation of hair follicles, external sources such as sweat or, in the case of newborns, amniotic fluid may also affect hair that has already emerged on the scalp [67, 71].

Further limitations are related to the psychological data assessment: Whereas the maternal steroids in the 3 cm hair segment reflected the last three months, only the perceived stress in the last month was considered as a covariate in the analyses (assessed with the PSS4, which instructs participants to refer to the past month). Furthermore, the covariate lifetime psychopathology was only assessed in self-report and not with a standardized psychiatric interview.

Strengths of this study are the assessment of steroids in hair as a cumulative measure of HPA axis activity, the consideration of two important steroids (cortisol and DHEA) and the investigation of hair samples of mother-newborn dyads extending existing literature on the CM-related transmission of HPA axis dysregulation, which most often confined analysis of steroid hormones to the offspring generation. Furthermore, the influence of parental behavior on steroid levels in the hair of newborns can be nearly excluded in this study, since hair samples were collected within six days after parturition. Remarkably, we observed an effect of CM on HPA axis activity in a sample of well-educated healthy women with relatively low psychosocial burden (only five women were worried about financial problems due to the child’s birth, four reported cramped living conditions [ratio of number of rooms/number of persons in the flat ≤0.5], and one woman experienced mild physical violence by her intimate partner within the last 8 weeks). However, results can thus only be generalized to a limited degree and studies on high-risk samples are needed.

## Implications for research and conclusion

Results of this study revealed an association of increasing cumulative DHEA, but not cortisol levels in maternal hair with rising number of CM experiences. This might reflect long-term alterations in the HPA axis in response to maltreatment experiences in childhood, which extend into pregnancy and highlights the importance to investigate DHEA in addition to cortisol. With due caution, our data also provide some support for the hypothesis that CM may influence the offspring’s prenatal HPA axis, as we observed a positive association of maternal CM and elevated DHEA concentrations in the newborns’ hair, but as a non-significant trend.

Measuring cumulative cortisol and DHEA concentrations in mothers and their newborns shortly after parturition seems promising for the investigation of prenatal biological effects of maternal trauma history on her offspring’s neuroendocrine system, without the confounding effect of parental behavior.

Further studies in larger (high-risk) samples are needed and would allow further analyses (e.g., taking ongoing life circumstances, time between CM and pregnancy, as well as the specific effects of different types of CM into account), in order to better understand the underlying biological mechanisms in the interplay between CM and a potentially altered HPA axis activity. Furthermore, longitudinal studies should investigate whether altered levels of DHEA in late gestation are related to altered patterns of HPA axis activity in childhood and adulthood. Pregnancy is associated with significant physiological alterations of HPA axis functioning and the biological function as well as implications of elevated DHEA in non-pregnant individuals cannot easily be transferred into the third trimester of pregnancy. Thus, long-term effects of increased DHEA levels in late gestation on health outcomes in the mothers and their offspring needs to be addressed in prospective studies and the investigation of hair DHEA levels in non-pregnant women with a history of CM seems warranted.

## Additional files

Additional file 1: Study procedure and drop-outs. Figure illustrating the study procedure (measurement points) and reporting drop-out rates and reasons for drop-out. (PDF 151 kb)

Additional file 2: Description of the data assessment using the MACE to assess adverse childhood experiences. Additional file 2 describes the procedure for the additional data assessment (i.e., a more detailed interview about adverse childhood experiences) with a subsample of mother-infant dyads three months postpartum. (PDF 152 kb)

Additional file 3: Summary of heteroscedastic regression analyses for variables predicting DHEA, and cortisol in hair samples of mothers and their newborns, who participated in three-months follow-up and were interviewed with the MACE. Table with results from heteroscedastic regression analyses for the subsample of participants described in Additional file 2. (PDF 132 kb)

Additional file 4: Regression line of maternal childhood maltreatment assessed by CTQ (left panel) and MACE (right panel) respectively and prenatal DHEA concentrations measured in newborn’s hair. The grey area shows the pointwise 95% confidence area. The figure illustrates the association of adverse childhood experiences (comparing two different instruments) and concentrations of DHEA in hair in a subsample of newborns (described in Additional file 2). (PDF 231 kb)

## Abbreviations

ACTH: Adrenocorticotropic hormone
AIC: Akaike’s Information Criterion
BMI: Body mass index
CM: Childhood maltreatment
CRH: Corticotropin-releasing hormone
CTQ: Childhood Trauma Questionnaire
DHEA: Dehydroepiandrosterone
HPA axis: Hypothalamic-pituitary-adrenal axis
M: Mean
MACE: Maltreatment and Abuse Chronology of Exposure
OLS: Ordinary least squares
PSS4: Perceived Stress Scale, 4-item version
PTSD: Posttraumatic stress disorder
SD: Standard deviation
