# Supplementary figures and images for: Alterations of hair cortisol and dehydroepiandrosterone in mother-infant-dyads with maternal childhood maltreatment
Source: BMC Psychiatry. 2017 Jun 6;17:213. doi: 10.1186/s12888-017-1367-2 (PMC5461775; doi:10.1186/s12888-017-1367-2)

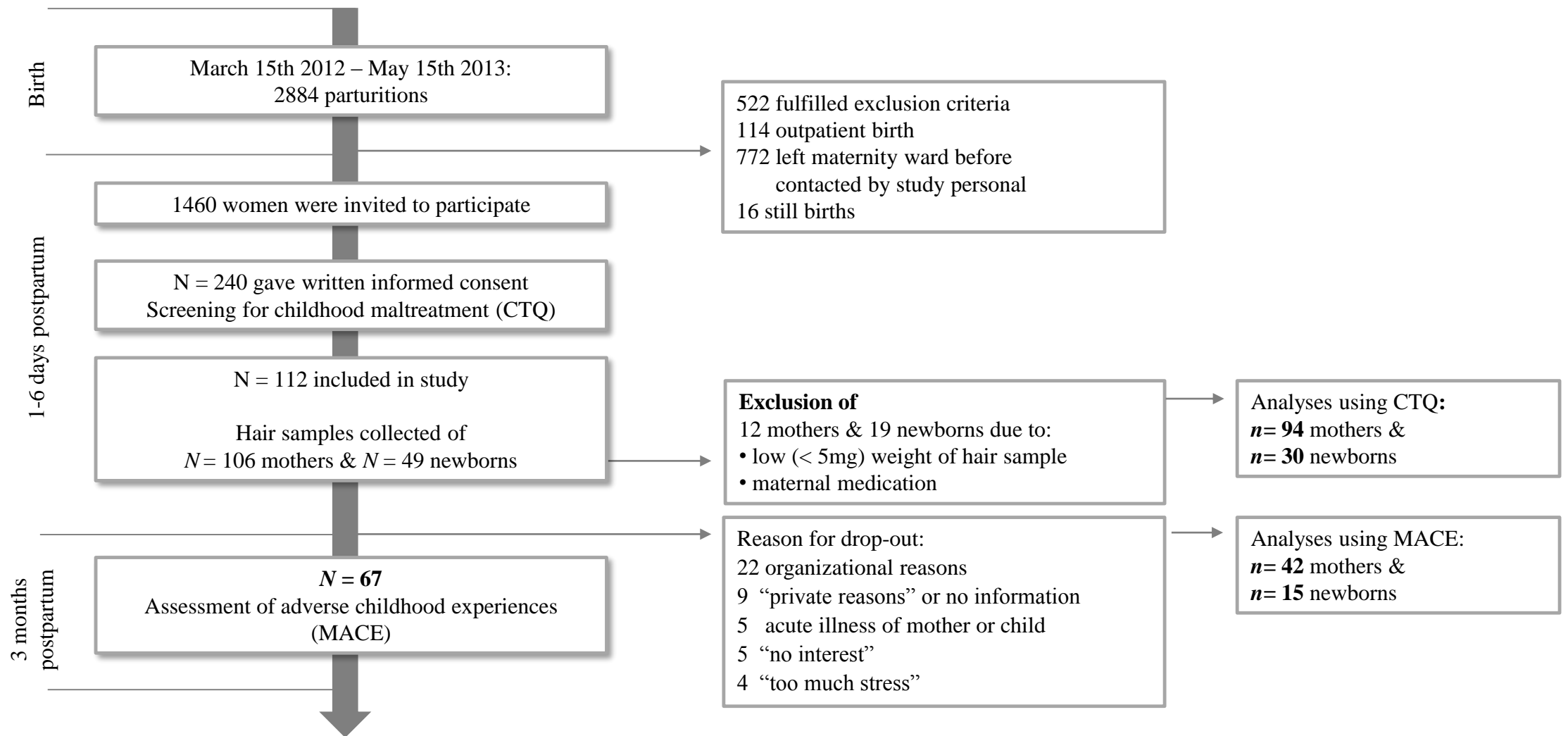

Supplement A. Study procedure and drop-outs.

Supplement: Supplementary file 1 — Study procedure and drop-outs. Figure illustrating the study procedure (measurement points) and reporting drop-out rates and reasons for drop-out. (PDF 151 kb) [file 12888_2017_1367_MOESM1_ESM.pdf]
