# Supplementary material for: Alterations of hair cortisol and dehydroepiandrosterone in mother-infant-dyads with maternal childhood maltreatment
Source: BMC Psychiatry. 2017 Jun 6;17:213. doi: 10.1186/s12888-017-1367-2 (PMC5461775; doi:10.1186/s12888-017-1367-2)
Supplement: Supplementary file 2 — Description of the data assessment using the MACE to assess adverse childhood experiences. Additional file 2 describes the procedure for the additional data assessment (i.e., a more detailed interview about adverse childhood experiences) with a subsample of mother-infant dyads three months postpartum. (PDF 152 kb) [file 12888_2017_1367_MOESM2_ESM.pdf]

Supplement B. Description of the data assessment using the MACE to assess adverse childhood experiences.

Three months postpartum (*mean* = 92 days; range = [77; 108] days) study participants were invited for a more detailed assessment of their adverse childhood experiences (cf. Supplement A). A total of 67 mothers participated and were interviewed by trained psychologists with the German version of the Maltreatment and Abuse Chronology of Exposure (MACE; in German: Belastende Kindheitserfahrungen [KERF]; Isele, D., Teicher, M. H., Ruf-Leuschner, M., Elbert, T., Kolassa, I.-T., Schury, K. & Schauer, M. 2014). A total of 75 statements about adverse childhood experiences were rated with “yes” or “no”. Ten subscales differentiate experiences of parental physical abuse, parental verbal abuse, parental nonverbal emotional abuse, sexual abuse, emotional neglect, physical neglect, the witness of physical violence towards parents, the witness of violence toward siblings, peer emotional violence, and peer physical violence. The MACE sum score accounts for the number of maltreatment events experienced (i.e., maltreatment load). Mothers participating at this three-month follow-up received an additional 40€ compensation.

Regression analyses using the MACE were restricted to the N = 42 mothers and N = 15 newborns who participated at three-month follow-up, provided hair samples of at least 5 mg and were not excluded due to maternal intake of medication which may influence HPA axis activity during pregnancy. For details on study procedure and drop-outs see Supplement A.
