# Supplementary material for: Alterations of hair cortisol and dehydroepiandrosterone in mother-infant-dyads with maternal childhood maltreatment
Source: BMC Psychiatry. 2017 Jun 6;17:213. doi: 10.1186/s12888-017-1367-2 (PMC5461775; doi:10.1186/s12888-017-1367-2)
Supplement: Supplementary file 3 — Summary of heteroscedastic regression analyses for variables predicting DHEA, and cortisol in hair samples of mothers and their newborns, who participated in three-months follow-up and were interviewed with the MACE. Table with results from heteroscedastic regression analyses for the subsample of participants described in Additional file 2. (PDF 132 kb) [file 12888_2017_1367_MOESM3_ESM.pdf]

Supplement C: Summary of heteroscedastic regression analysis for variables predicting DHEA, and cortisol in hair samples of mothers and their newborns, who participated at three-months follow-up and were interviewed with the MACE.

Maternal hair data

**DHEA concentrations (*N* = 41[†])**	***b***	***SE***	***t (39)***	***p***
Childhood maltreatment (MACE sum score)	0.13	0.10	1.40	.169
Age	0.68	0.25	2.66	.012*
Perceived stress (PSS4)	0.27	0.42	0.59	.556
Batch effect	3.82	2.30	1.67	.104
R^2 = .38, F(4, 36) = 4.88, p = .006**				

**Cortisol concentrations (*N* = 42)**	***b***	***SE***	***t(40)***	***p***
Childhood maltreatment (MACE sum score)	-0.02	0.25	0.06	.959
R^2 = .04, F(1,40) = 0.36, p = .556				

Newborn's hair data

**DHEA concentrations (*N* = 15)**	***b***	***SE***	***t(13)***	***p***
Childhood maltreatment (MACE sum score)	0.07	0.03	3.01	.010**
R^2 = .44, F(1, 13) = 10.21, p = .007**				

**Cortisol concentrations (*N* = 15)**	***b***	***SE***	***t(13)***	***p***
Childhood maltreatment (MACE sum score)	2.35	1.56	1.63	.132
Maternal age	-15.24	4.51	-3.45	.004
R^2 = .65, F(2, 12) = 12.29, p = .001**				

* < .05, ** < .01, *** <. 001

[†] Excluding outlier in DHEA score

MACE = Maltreatment and Abuse Chronology of Exposure; PSS4 = four-item version of the Perceived Stress Scale
