# Supplementary material for: Alterations of hair cortisol and dehydroepiandrosterone in mother-infant-dyads with maternal childhood maltreatment
Source: BMC Psychiatry. 2017 Jun 6;17:213. doi: 10.1186/s12888-017-1367-2 (PMC5461775; doi:10.1186/s12888-017-1367-2)
Supplement: Supplementary file 4 — Regression line of maternal childhood maltreatment assessed by CTQ (left panel) and MACE (right panel) respectively and prenatal DHEA concentrations measured in newborn’s hair. The grey area shows the pointwise 95% confidence area. The figure illustrates the association of adverse childhood experiences (comparing two different instruments) and concentrations of DHEA in hair in a subsample of newborns (described in Additional file 2). (PDF 231 kb) [file 12888_2017_1367_MOESM4_ESM.pdf]

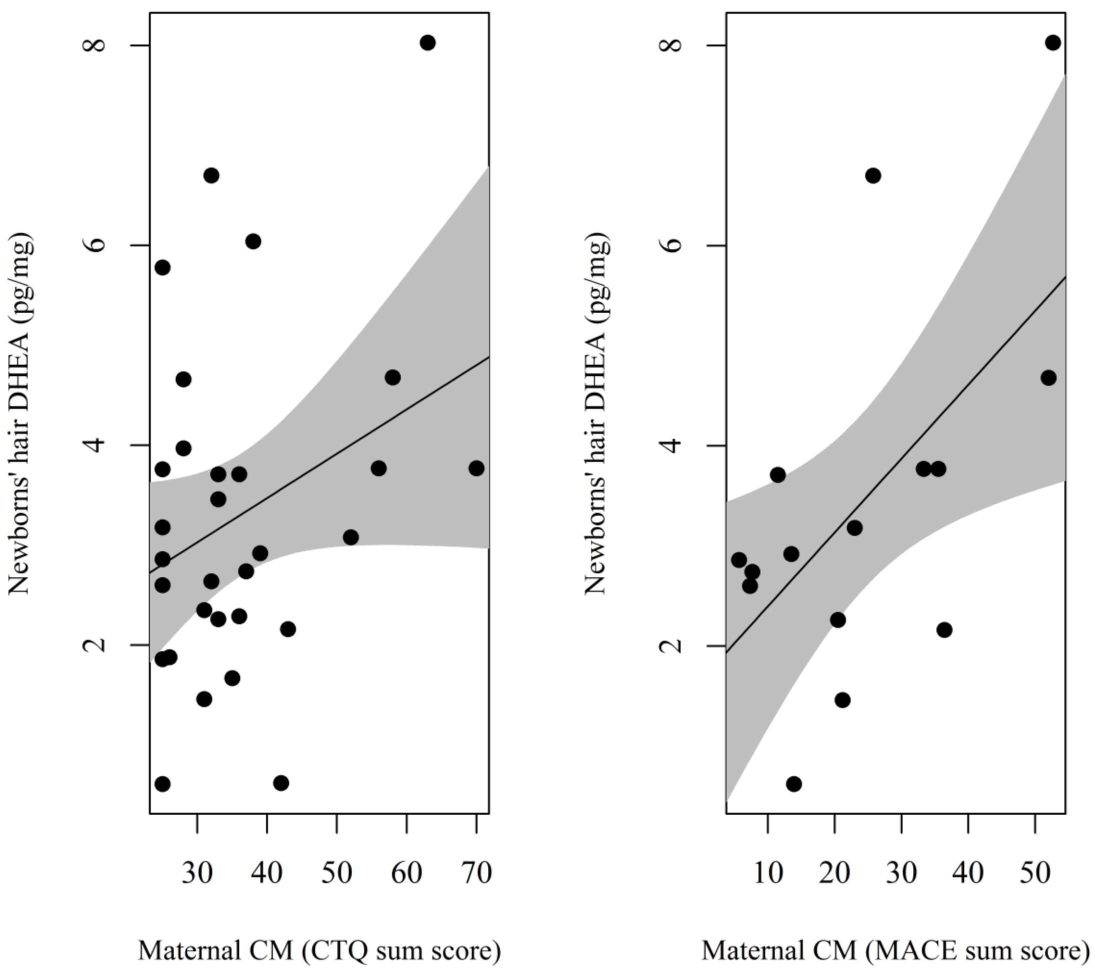

Supplement D. Regression line of maternal childhood maltreatment assessed by CTQ (left panel) and MACE (right panel) respectively and prenatal DHEA concentrations measured in newborn's hair. The grey area shows the pointwise 95% confidence area.
